# Frecuencia del trauma genitourinario en accidentes de tránsito en motocicleta: Revisión de alcance

**DOI:** 10.15446/rsap.V26n4.115133

**Published:** 2024-07-01

**Authors:** Gabriela Castañeda-Millán, David A. Hernández-Aparicio, David Castañeda-Millán, Javier H. Eslava-Schmalbach

**Affiliations:** 1 GC: MD. Facultad de Medicina, Universidad Nacional de Colombia. Bogotá, Colombia. gacastanedami@unal.edu.co Universidad Nacional de Colombia Facultad de Medicina Universidad Nacional de Colombia Bogotá Colombia gacastanedami@unal.edu.co; 2 DH: MD. Facultad de Medicina, Universidad Nacional de Colombia. Bogotá, Colombia. davahernandezapa@unal.edu.co Universidad Nacional de Colombia Facultad de Medicina Universidad Nacional de Colombia Bogotá Colombia davahernandezapa@unal.edu.co; 3 DC: MD. Uról. M. Sc. Donación y Trasplantes. Facultad de Medicina, Universidad Nacional de Colombia. Bogotá, Colombia. dacastanedam@unal.edu.co Universidad Nacional de Colombia Facultad de Medicina Universidad Nacional de Colombia Bogotá Colombia dacastanedam@unal.edu.co; 4 JE: MD. Anest. M. Sc. Epidemiología Clínica. Docente. Facultad de Medicina, Universidad Nacional de Colombia. Bogotá, Colombia. jheslavas@unal.edu.co Universidad Nacional de Colombia Facultad de Medicina Universidad Nacional de Colombia Bogotá Colombia jheslavas@unal.edu.co

**Keywords:** Accidentes de tránsito, motocicletas, adulto, sistema urogenital, genitales masculinos, genitales femeninos *(fuente: DeCS, BIREME)*, Accidents traffic, motorcycles, urogenital system, adult, genitalia male, genitalia female *(source: MeSH, NLM)*

## Abstract

**Objetivo:**

Describir la frecuencia del trauma genitourinario (TGU) causado por accidentes de tránsito en motocicleta.

**Metodología:**

Revisión de alcance de acuerdo con la metodología propuesta por Arksey y O'Malley y la extensión para revisiones de alcance Prisma. Las búsquedas se realizaron en PubMed y Embase. Se incluyeron todos los estudios que describieron la frecuencia del TGU en población adulta (> 18 años) como consecuencia de accidentes de tránsito en motocicleta. Dos revisores independientes se encargaron de la selección por título y resumen y posteriormente de la evaluación de la elegibilidad y la calidad de los estudios en texto completo.

**Resultados:**

La frecuencia de TGU fue mayor en hombres, con cifras entre el 94,5% y el 96,5%, respecto a 3,4% y 5,5% en mujeres. Los conductores fueron los más afectados en comparación con los pasajeros, con una proporción de 96,4%. Los testículos fueron los principales órganos afectados, con frecuencias de trauma reportadas entre el 0,4% y el 41%, seguidos del riñón (2,4% a 35%); escroto (0% a 14%); pene (0% a 13%); vejiga (0,4% a 4%); uréter (0 a 0,02%); uretra (0,2% a 2%); vagina (1%) y vulva (1%).

**Conclusión:**

La frecuencia del TGU en pacientes involucrados en accidente de motocicleta es alta. Afecta con mayor frecuencia a los hombres jóvenes conductores de motocicleta; los órganos más afectados son los genitales externos masculinos, los riñones y la vejiga. No obstante, en mujeres, es una causa importante de TGU no obstétrico.

## Abstract (en)

**Objective:**

To describe the frequency of genitourinary trauma (GUT) caused by motorcycle traffic accidents.

**Methodology:**

Scoping review conducted according to the methodology proposed by Arksey and O'Malley and the Prisma extension for scoping reviews. Searches were conducted in PubMed and Embase. All studies that described the frequency of GUT in adults (>18 years) as a result of motorcycle traffic accidents were included. Two independent reviewers were responsible for the selection based on titles and abstracts and subsequently evaluated the eligibility and quality of the studies using full-text articles.

**Results:**

The frequency of GUT was higher in men, ranging from 94.5% to 96.5%, compared to 3.4% to 5.5% in women. Drivers were more affected than passengers, with a proportion of 96.4%. The testicles were the primary organs affected, with reported trauma frequencies ranging from 0.4% to 41%, followed by the kidney (2.4% to 35%), scrotum (0% to 14%), penis (0% to 13%), bladder (0.4% to 4%), ureter (0% to 0.02%), urethra (0.2% to 2%), vagina (1%), and vulva (1%).

**Conclusion:**

The frequency of gut in patients involved in motorcycle accidents is high. It primarily affects young male motorcycle drivers; the most affected organs are the male external genitalia, kidneys, and bladder. However, in women, it is a significant cause of non-obstetric gut.

Los accidentes de tránsito son un problema de salud pública evitable que ha venido en aumento en los últimos años 1. A nivel global, son una de las primeras causas de mortalidad en niños, adolescentes y adultos jóvenes 2. La Organización Mundial de la Salud (OMS) ha informado que cada año los accidentes de tránsito ocasionan 1,29 millones de muertes y 50 millones de lesiones 2, con una alta carga de enfermedad, económica y social derivada de las muertes anticipadas y la discapacidad asociada 2.

En el caso particular de los países en desarrollo, se ha identificado una tendencia al incremento en los accidentes de tránsito, principalmente en aquellos que involucran motocicletas 3, dado que su uso ha aumentado, como alternativa para las personas en desventaja socioeconómica, lo que les permite superar las brechas en movilidad y las inequidades en el acceso a los servicios de transporte público 4,5.

El trauma craneoencefálico es el tipo de trauma más frecuente que se presenta en las víctimas de accidentes de tránsito en motocicleta 6, seguido del trauma de tórax y de abdomen que son los responsables de hasta el 25% de estos fallecimientos 6. Además, los accidentes de tránsito representan una de las principales causas de trauma urológico. Aquellos que implican trauma abdominal y trauma pélvico suelen asociarse con TGU, presente en aproximadamente el 10% de estos casos. Esta situación contribuye al aumento de la morbilidad y mortalidad en los pacientes afectados 7.

El TGU se divide en tres regiones anatómicas: el tracto superior formado por ríñones y uréteres, el tracto inferior que consta de la vejiga y la uretra, y los genitales externos que son el pene, el escroto, y los testículos en hombres, y, la vulva en mujeres 8. El trauma por accidentes de tránsito puede ocurrir en todos los niveles. En general, el trauma renal es el más frecuente causado por trauma contundente o mecanismos de aceleración y desaceleración 9. El trauma de vejiga frecuentemente ocurre concomitante con fractura de pelvis 10. Y, finalmente, el trauma en los genitales externos, principalmente aquellos que involucran los testículos y el escroto, también se han asociado con mecanismos de fuerza externa donde la primera causa son los accidentes de tránsito 11.

Por esta razón, aunque el objetivo principal de la evaluación de los pacientes con trauma es la estabilización de las lesiones que comprometen la vida, se deben diagnosticar y tratar las lesiones del tracto genitourinario 12. Si bien es infrecuente que este tipo de trauma ponga en peligro la vida, puede desencadenar complicaciones asociadas y secuelas irreversibles, que podrían prevenirse mediante un diagnóstico y un tratamiento tempranos 12,13. Se ha descrito que no considerar el potencial TGU conlleva retrasos en el diagnóstico que finalmente se traducen en una morbilidad significativa para los pacientes, ya que pueden surgir complicaciones que generan daños fisiológicos, psicológicos, sexuales y reproductivos 12,14. Además, se debe tener en cuenta que el impacto social y económico puede ser mayor, ya que los más afectados por este tipo de lesiones son los hombres jóvenes con un nivel socioeconómico bajo, quienes tienen un mayor riesgo de sufrir algún tipo de trauma 15.

Por las razones expuestas, la caracterización adecuada del TGU en pacientes que sufren accidentes de tránsito en moto permite sospechar, diagnosticar y tratar a los pacientes politraumatizados con lesiones urológicas de forma adecuada, evitando complicaciones, morbilidad y mortalidad asociadas 16. En ese sentido, el objetivo de esta revisión es describir la frecuencia del TGU y sus diferentes categorías, relacionadas con accidentes de tránsito en moto.

## METODOLOGÍA

Este estudio es una revisión de alcance de acuerdo con la metodología propuesta por Arksey y O'Malley 17, y las directrices para revisiones sistemáticas y metaanálisis Prisma, en su extensión para revisiones de alcance, y el informe se presenta de acuerdo con esta lista de verificación 18.

### Criterios de inclusión

Se incluyeron investigaciones originales sin establecer un límite de tiempo o filtro de idiomas. Se incluyeron todos los estudios que describieron la frecuencia del TGU en población adulta (≥ 18 años) como consecuencia de accidentes de tránsito en motocicleta.

### Criterios de exclusión

Se excluyeron reportes de caso, pósteres, cartas al editor y revisiones de la literatura. Además, se excluyeron estudios en población pediátrica (≤ 18 años) y aquellos considerados como no relacionados, ya que describían otro tipo de lesiones, diferentes a las genitourinarias.

También se excluyeron estudios que describían la frecuencia de las causas de TGU, entre las cuales se identificaban los accidentes de tránsito en motocicleta; sin embargo, no respondían a la pregunta de investigación, ya que no mostraban la ocurrencia de lesiones urológicas específicamente en contexto.

### Estrategia de búsqueda

Se realizaron búsquedas en las bases de datos PubMed y Embase. Las estrategias de búsqueda se construyeron en consenso con los cuatro autores, el detalle de la estrategia y sus respectivos términos específicos indexados para cada base de datos se describen con detalle en la Tabla 1.

**Tabla 1:** Estrategia de búsqueda

Estrategia de búsqueda empleada en Pubmed
((((("Adult"[MeSH Terms] OR "Middle Aged"[MeSH Terms] OR "Young Adult"[MeSH Terms] OR "Aged"[MeSH Terms]) AND "accidents, traffic"[MeSH Terms]) OR "Traffic Accidents"[All Fields]) AND "Motorcycles"[MeSH Terms]) OR "Motorcycle"[All Fields] OR "Motorbike"[All Fields] OR "Motorcycle Accidents"[All Fields] OR "Motorbike Accidents"[All Fields]) AND ("urogenital system/injuries"[MeSH Terms] OR "genitalia, female/injuries"[MeSH Terms] OR "genitalia, male/injuries"[MeSH Terms] OR "penis/injuries"[MeSH Terms] OR "prostate/injuries"[MeSH Terms] OR "scrotum/injuries"[MeSH Terms] OR "Testis"[MeSH Terms] OR "urinary tract/injuries"[MeSH Terms] OR "kidney/injuries"[MeSH Terms] OR "ureter/injuries"[MeSH Terms] OR "urethra/injuries"[MeSH Terms] OR "urinary bladder/injuries"[MeSH Terms] OR "Genitourinary Trauma"[All Fields] OR "Genitourinary Injuries"[All Fields] OR "Urogenital Trauma"[All Fields] OR "Urogenital injuries"[All Fields] OR "Genitalia Trauma"[All Fields] OR "Genitalia injuries"[All Fields] OR "Urinary Tract Trauma"[All Fields] OR "urinary tract/injuries"[All Fields] OR "Urological Trauma"[All Fields] OR "Urotrauma"[All Fields])
Estrategia de búsqueda empleada en Embase
('adult'/exp OR 'adult' OR 'adults' OR 'grown-ups' OR 'grownup' OR 'grownups' OR 'aged'/exp OR 'aged' OR 'aged patient' OR 'aged people' OR 'aged person' OR 'aged subject' OR 'elderly' OR 'elderly patient' OR 'elderly people' OR 'elderly person' OR 'elderly subject' OR 'senior citizen' OR 'senium' OR 'middle aged'/exp OR 'middle age' OR 'middle aged' OR 'young adult'/exp OR 'adult, young' OR 'prime adult' OR 'prime adults' OR 'young adult' OR 'young adults') AND ('traffic accident'/exp OR 'accident traffic' OR 'accident, road' OR 'accident, streetcar' OR 'accident, traffic' OR 'accidents, traffic' OR 'automobile accident' OR 'automobile collision' OR 'car accident' OR 'motor vehicle accident' OR 'motorcar accident' OR 'motorcycle accident' OR 'road accident' OR 'streetcar accident' OR 'traffic accident' OR 'traffic accidents' OR 'vehicle accident' OR 'vehicular accident') AND ('motorcycle'/exp OR 'motorbike' OR 'motorcycle' OR 'motorcycles' OR 'motorised bicycle' OR 'motorized bicycle' OR 'two-wheeled motor vehicle') AND ('urogenital tract injury'/exp OR 'urogenital injuries' OR 'urogenital injury' OR 'urogenital tract injury' OR 'urogenital trauma' OR 'genital injury'/exp OR 'genital injuries' OR 'genital injury' OR 'genital tract injuries' OR 'genital tract injury' OR 'genital tract trauma' OR 'genital tract traumas' OR 'genital trauma' OR 'genital traumas' OR 'genital wound' OR 'urinary tract injury'/exp OR 'urinary tract injury' OR 'urinary tract trauma' OR 'urogenital tract rupture'/exp OR 'urogenital tract rupture' OR 'penis injury'/exp OR 'cavernosal rupture' OR 'cavernous body rupture' OR 'corpus cavernosum rupture' OR 'penile fracture' OR 'penile fractures' OR 'penile injuries' OR 'penile injury' OR 'penile trauma' OR 'penile traumas' OR 'penis fracture' OR 'penis injury' OR 'penis trauma' OR 'testis injury'/exp OR 'injury, testis' OR 'testicle injuries' OR 'testicle injury' OR 'testicle trauma' OR 'testicular injuries' OR 'testicular injury' OR 'testicular trauma' OR 'testis damage' OR 'testis injuries' OR 'testis injury' OR 'testis rupture' OR 'testis trauma' OR 'trauma, testis' OR 'vaginal injury'/exp OR 'vagina injury' OR 'vagina trauma' OR 'vaginal injuries' OR 'vaginal injury' OR 'vaginal trauma' OR 'vaginal traumas' OR 'vulvar injuries' OR 'vulvar injury' OR 'vulvar trauma' OR 'vulvar traumas' OR 'bladder injury'/exp OR 'bladder damage' OR 'bladder injury' OR 'bladder lesion' OR 'bladder trauma' OR 'bladder wall lesion' OR 'urinary bladder trauma' OR 'kidney injury'/exp OR 'acute renal injury' OR 'chronic kidney injury' OR 'chronic renal injury' OR 'kidney cortex lesion' OR 'kidney damage' OR 'kidney injury' OR 'kidney lesion' OR 'kidney trauma' OR 'renal damage' OR 'renal injury' OR 'renal lesion' OR 'renal trauma' OR 'trauma, kidney' OR 'trauma, renal' OR 'ureter injury'/exp OR 'injury, ureter' OR 'trauma, ureter' OR 'trauma, ureteral' OR 'uretal injury' OR 'ureter injury' OR 'ureter trauma' OR 'ureteral injury' OR 'ureteral trauma' OR 'urethra injury'/exp OR 'urethra injury' OR 'urethra lesion' OR 'urethra trauma' OR 'urethral injury' OR 'urethral trauma')

### Selección de artículos

Dos revisiones independientes (GC y DH) hicieron el tamizaje por título y resumen de todos los artículos recuperados de las búsquedas y evaluaron su elegibilidad según los criterios de inclusión. Se recuperaron todos los artículos que cumplían con los criterios de inclusión en texto completo y dos revisores independientes (GC y DH) leyeron estos artículos para evaluar la elegibilidad. Los desacuerdos entre los revisores se resolvieron por consenso. La selección de los artículos se llevó a cabo utilizando la plataforma RAYYAN QCRI®.

### Evaluación de calidad

Se utilizó la lista de chequeo STROBE para artículos observacionales, a fin de evaluar la calidad del reporte de los estudios (19). Dos revisores (GC y DH) hicieron esta evaluación de forma independiente. Se evaluaron los 22 ítems de la lista de chequeo y se estandarizaron los resultados de la evaluación de la siguiente forma: calidad alta: (8/10 =18 ítems o más), calidad moderada (6-8/10 =13-17 ítems) y calidad baja (< 6/10 =menos de 13 ítems).

### Extracción y síntesis de los datos

Un revisor (GC) extrajo los datos de las revisiones incluidas y otro revisor (DH) verificó la exactitud de la extracción. Se utilizó un formulario de extracción de datos diseñado para extraer de forma independiente las características de los estudios, así como la información correspondiente al sexo, la categoría de usuario, la categoría y la frecuencia del TGU en los pacientes que sufrieron accidentes de tránsito en moto. De la misma forma que en el proceso de selección, los desacuerdos se resolvieron por consenso.

## RESULTADOS

**Figura 1:**
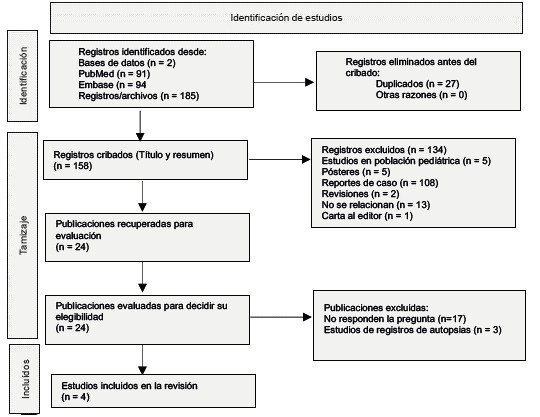
Flujograma Prisma del proceso de selección de artículos

En total, se identificaron 185 estudios. Tras eliminar 27 duplicados, se tamizaron 158 por título y resumen, se evaluaron 24 estudios en texto completo para decidir su elegibilidad. Finalmente, se incluyeron 4 publicaciones, mientras que 20 estudios fueron excluidos (Figura 1).

Los estudios incluidos se llevaron a cabo en Estados Unidos (n=1), Francia (n=2) y Reino Unido (n=1). Todos los estudios fueron observacionales de cohorte, 3 de ellos fueron retrospectivos y uno fue prospectivo. Los artículos de Terrier *et al.*^21^ Moss *et al.*^22^ y Song *et al.*^23^ fueron de alta calidad.

Terrier *et al.* identificaron 162 690 pacientes que sufrieron accidentes de tránsito en Ródano (Francia) entre 1996 y 2013. La edad promedio de los pacientes fue de 28 años. De esta cohorte 35 929 fueron motociclistas, de los cuales el 1,3% (n=453) tuvo TGU; más de la mitad (56%) de estos últimos requirió hospitalización 21.

En el mismo estudio, el 62% de lesiones urológicas en usuarios de motocicleta se localizaron en los genitales externos. La frecuencia de trauma en escroto, testículos y pene fue mayor en comparación con conductores de automóvil, ciclistas y peatones. Además, la proporción de trauma renal grado V fue mayor en motociclistas (2%), respecto a los demás usuarios (0-1%). Además, se observó que el TGU fue más frecuente en el grupo de 26 a 35 años, y se identificó que ser hombre (OR 2,8; IC 95%: 1,9-4,3) y presentar el accidente en áreas rurales (OR 1,7; IC 95%: 1,4-2,3) son factores de riesgo para presentar TGU en motociclistas 21.

Moss *et al.* identificaron 12 374 pacientes que sufrieron accidentes de tránsito en motocicleta entre 2012 y 2016 en Reino Unido. De estos, 11 924 fueron conductores y 450 pasajeros. El 6% de estos pacientes tuvieron TGU. La mediana de edad fue de 34 años en conductores y 24 años en pasajeros. Además, Moss *et al.* reportaron que el Injury Severity Score (ISS) en los motociclistas con TGU (Me=27 en conductores y Me=25 en pasajeros) fue mayor en comparación con los que no tuvieron TGU (Me=13 conductores y pasajeros). Así mismo, identificaron que la mortalidad fue más alta en conductores de motocicleta (9,5%), respecto a 3,2% en conductores sin TGU 22.

Song *et al.* identificaron 3 137 799 pacientes con trauma en Estados Unidos entre 2017 y 2019, de los cuales 6 897 estuvieron involucrados en accidentes de tránsito en motocicleta. La lesión cerrada de uretra en mujeres ocurrió en el 0,2% (n=14) de los afectados. Además, se observó que los accidentes de tránsito en motocicleta incrementaron el riesgo de este tipo de lesión hasta 7 veces (OR 6,92, p<0,001) 23.

Paparael *et al.* hicieron el análisis de 43 056 víctimas de accidentes de tránsito en Ródano (Francia) entre 1996 y 2001. Se reportaron 199 (0,46%) casos de TGU, 152 hombres y 47 mujeres, con una edad promedio de 30,3 años. Los usuarios de motocicleta con TGU todos fueron hombres (n=78). En el 64% de los casos, la lesión se localizó en los genitales externos, con mayor frecuencia en los testículos 24.

En los dos estudios en que se incluyeron ambos sexos, la frecuencia de trauma fue mayor en hombres, con cifras entre el 94,5% y el 96,5% respecto a 3,5% y 5,5% en mujeres. Los conductores fueron los más afectados en comparación con los pasajeros, con una proporción de 97,6% frente a 2,4%.

Los testículos fueron los principales órganos afectados, con frecuencias de trauma reportadas entre el 0,4% y el 41%. El siguiente órgano más frecuentemente afectado fue el riñón, con frecuencias reportadas entre el 2,4% y el 35% de los pacientes, seguido del escroto (0% al 14%), el pene (0% al 13%), la vejiga (0,4% al 4%), el uréter (0 al 0,02%), la uretra (0,2% al 2%), la vagina (1%) y la vulva (1%). Las medidas de frecuencia identificadas y los detalles se presentan en la Tabla 2.

**Tabla 2:** Estudios que incluyeron medidas de frecuencia de TGU causado por accidentes de tránsito en motocicleta, según la categoría de trauma

Primer autor, año	País	Tipo de estudio	Calidad*	Sexo %	Categoría de usuario %	Categoría de trauma	Frecuencia %
Terrier 21	Francia	Cohorte retrospectiva (n=453)	Alta	Hombres 94,5(n=428) Mujeres 5,5 (n=25)	No informa	Testículos	38 (n=170)
						Riñón	35 (n=157)
						Escroto	14 (n=62)
						Pene	8 (n=37)
						Vejiga	4 (n=17)
						Uretra	2 (n=7)
						Periné	1 (n=6)
						Vagina	1 (n=4)
						Vulva	1 (n=4)
						Uréter	0 (n=1)
Moss 22	Reino Unido	Cohorte prospectiva (n=745)	Alta	Hombres 96,1(n=716) Mujeres 3,9 (n=29)	Conductores 97,6 (n=727) Hombres 96,8 (n=704) Mujeres 3,2 (n=23)	Riñón	4 (n=477)
						Vejiga	0,4 (n=48)
						Testículos	0,4 (n=48)
						Escroto	0,3 (n=32)
						Pene	0,3 (n=31)
						Uretra	0,2 (n=26)
						Periné	0,06 (n=7)
						Uréter	0,02 (n=3)
					Pasajeros 2,4 (n=18) Hombres 83,3 (n=15) Mujeres 16,7 (n=3)	Riñón	2,4 (n=11)
						Vejiga	0,7 (n=3)
						Testículos	0,7 (n=3)
						Uretra	0,2 (n=1)
						Pene	0
						Periné	0
						Escroto	0
						Uréter	0
Paparel 24	Francia	Cohorte retrospectiva (n=78)	Baja	Hombres (100%)	No informa	Testículos	41
						Riñón	28
						Pene	13
						Escroto	10
Song 23	Estados Unidos	Cohorte retrospectiva (n=6 897)	Alta	Mujeres	No informa	Uretra	0,2 (n=14)

Fuente: Nota: ^*^ Se utilizó la lista de chequeo STROBE para evaluar la calidad del reporte de los estudios.

## DISCUSIÓN

En esta revisión se logró identificar cuatro estudios que describieron la frecuencia de TGU causados por accidentes de tránsito en motocicleta. Todos fueron llevados a cabo en países de ingresos altos a partir de datos obtenidos de registros de trauma. Además, se encontró que, en este contexto, el TGU es entre 17 y 28 veces más frecuente en hombres que en mujeres, especialmente entre los 25 y los 35 años 21,22, y la ocurrencia de TGU fue hasta 40 veces mayor en conductores que en pasajeros 22.

Se identificaron diferencias en la frecuencia del trauma urológico según el tipo de usuario, de tal manera que el trauma renal fue más frecuente en conductores, mientras que el trauma de vejiga y de testículos fue más frecuente en pasajeros 22. Además, la mortalidad fue 3 veces más alta en los conductores de motocicleta con TGU en comparación con los que los no tuvieron TGU y el *Injury Severity Score* (ISS) en los pacientes con TGU dobló la cifra de los que no tuvieron TGU 22, lo cual sugiere que, si bien la incidencia de estas lesiones es baja, las secuelas resultantes pueden ser graves.

Según la región anatómica afectada, en los pacientes que tuvieron accidentes en motocicleta se encontró una frecuencia de trauma renal que osciló entre 2,4% y 35%, superior a la reportada previamente del 5% para todas las causas de trauma 10. Además, el trauma renal fue más grave en motociclistas. Terrier et al, informaron que la proporción de trauma renal grado V fue mayor en motociclistas que en los demás usuarios de las vías (conductores de automóvil, ciclistas y peatones) 21. Así mismo, en los genitales externos se identificó una frecuencia más alta para el trauma de testículos, que alcanzó cifras hasta del 41%, seguido del trauma de escroto que se reportó hasta en el 14% y el trauma de pene que se presentó hasta en el 13% de los casos. Estas cifras superaron la incidencia informada de menos del 1% de estas lesiones por todas las causas de trauma, pues en general se considera que las lesiones en los genitales externos son raras debido a que se encuentran protegidos por su posición anatómica 25.

Finalmente, el trauma de vejiga ocurrió hasta en el 4% de los casos, ligeramente superior a lo reportado previamente por Bjurlin *et al.,* quienes informaron una prevalencia del 3,6% de trauma vesical en pacientes con fractura pélvica 26. De otra parte, el trauma de uretra y uréteres fue menos frecuente, con estimaciones de 2% y 0,02%, respectivamente. La frecuencia de estas lesiones se encontró dentro de lo esperado, con cifras inferiores al 4% y al 1%, respectivamente, por otros autores 27.

Un hallazgo importante fue que todos los estudios se llevaron a cabo en países de ingresos altos. Sin embargo, el trauma en general tiene una mayor carga de enfermedad, económica y social en países de ingresos medios y bajos 28. Además, los accidentes de tránsito y particularmente aquellos que involucran motociclistas tienen un mayor impacto y ocurren con mayor frecuencia en países en desarrollo, en los que la mortalidad por este tipo de accidentes se ha incrementado en los últimos años (5,2931). No obstante, a pesar de esta realidad, en esta revisión no se identificaron estudios que permitieran estimar la frecuencia del TGU por accidentes de tránsito en motocicleta en estos países.

Los cuatro estudios fueron realizados a partir de registros de trauma. Esto es fundamental ya que el desarrollo de registros de trauma es una estrategia para mejorar la calidad de atención de los pacientes que sufren lesiones 32. En los países desarrollados se han implementado estos registros en centros de trauma, con el fin de recolectar de forma sistemática los datos de los pacientes lesionados, lo que ha permitido hacer un seguimiento que ha mejorado los desenlaces clínicos en países de altos ingresos 33, en los que se ha logrado una reducción de la mortalidad y la discapacidad asociada 34. No obstante, los países de bajos y medianos ingresos han sido excluidos de la implementación de estos registros ya que enfrentan barreras como recursos limitados, la calidad de los datos, la infraestructura, entre otros 32,33.

Por otra parte, se identificaron brechas por sexo, Terrier *et al.* identificaron que los hombres tuvieron 3 veces más riesgo de sufrir lesiones urológicas en accidentes de tránsito en motocicleta 21. Sin embargo, se debe mencionar que las lesiones específicamente en mujeres solo se describieron en dos estudios. En uno de ellos se identificó que el 1% de las pacientes presentó trauma de vulva y de vagina secundario a este tipo de accidentes 21, mientras que en el segundo se identificó que el trauma uretral en mujeres se presentó en el 0,2% de todos los pacientes que estuvieron involucrados en accidentes de tránsito en motocicleta 23.

Si bien esta disparidad puede deberse a un mayor impacto en los hombres, ya que se ha descrito que son los más afectados en los accidentes y la mayor cantidad de usuarios de motocicleta 3, es necesario desarrollar estudios que permitan describir las lesiones urológicas en mujeres ya que con la información disponible no es posible comprender el impacto de estas lesiones en esta población. Además, Song *et al.* identificaron que las lesiones urológicas, como las lesiones uretrales en mujeres, son lesiones centinela que actúan como marcadores de lesiones más graves que pueden llevar a la muerte 23.

Este estudio pone de manifiesto el vacío en el conocimiento existente respecto a estas lesiones y según lo reportado expone que la frecuencia de TGU es más alta en pacientes que sufren accidentes de tránsito en motocicleta. Es el primero que revisa la frecuencia de TGU relacionado con accidentes de tránsito en motocicleta. Sin embargo, es importante mencionar como limitación que no se encontraron estudios realizados en Latinoamérica y el Caribe, donde la incidencia de accidentes de tránsito en motocicleta es significativamente mayor 5. Por lo tanto, estos resultados no pueden extrapolarse directamente a esta población.

Además, es importante tener en cuenta que hay una mayor documentación de trauma urológico en hombres, mientras que las lesiones en mujeres están subrepresentadas en la literatura científica. Es fundamental abordar este tema, ya que las lesiones urológicas pueden incrementar la gravedad de las lesiones, la mortalidad y se pueden considerar lesiones centinela que son marcadores de lesiones más graves en los pacientes.

En general, la frecuencia del TGU en pacientes involucrados en accidente de motocicleta es alta. Afecta con mayor frecuencia a los hombres jóvenes conductores de motocicleta y los órganos más afectados son los genitales externos masculinos, los riñones y la vejiga. No obstante, en mujeres es una causa importante de TGU no obstétrico.

En ese sentido, es fundamental considerar la posibilidad de TGU en los pacientes que sufren accidentes, ya sea como conductores o pasajeros de motocicletas. A menudo, estas posibles lesiones tienden a subestimarse en pacientes politraumatizados, ya que no es usual que comprometan la vida. Sin embargo, debido a su alta frecuencia en los accidentes de tránsito que involucran motocicletas, se debe prestar especial atención al examen genital en estos pacientes. El hecho de pasar por alto el diagnóstico y el tratamiento de estas lesiones puede resultar en complicaciones y morbilidades potenciales, que no son abordadas en el alcance de este estudio.

Adicionalmente, resulta necesario establecer registros de trauma en los países de bajos y medianos ingresos, ya que el seguimiento a los pacientes lesionados puede mejorar los desenlaces clínicos de estos pacientes. Además, la recopilación y el análisis de este tipo de lesiones permitiría una comprensión más completa del TGU en países en desarrollo. Esta medida es esencial dado el creciente uso de la motocicleta como medio de transporte en estos países. Por otra parte, se sugiere prestar una mayor atención y documentar más exhaustivamente las lesiones urológicas no obstétricas en mujeres en futuras investigaciones ♦
